# Identification of Small Molecules That Suppress Ricin-Induced Stress-Activated Signaling Pathways

**DOI:** 10.1371/journal.pone.0049075

**Published:** 2012-11-01

**Authors:** Paul G. Wahome, Sarita Ahlawat, Nicholas J. Mantis

**Affiliations:** 1 Division of Infectious Diseases, Wadsworth Center, New York State Department of Health, Albany, New York, United States of America; 2 Department of Biomedical Sciences, University at Albany School of Public Health, Albany, New York, United States of America; Institut Curie, France

## Abstract

Ricin is a member of the ribosome-inactivating protein (RIP) family of plant and bacterial toxins. In this study we used a high-throughput, cell-based assay to screen more than 118,000 compounds from diverse chemical libraries for molecules that reduced ricin-induced cell death. We describe three compounds, PW66, PW69, and PW72 that at micromolar concentrations significantly delayed ricin-induced cell death. None of the compounds had any demonstrable effect on ricin's ability to arrest protein synthesis in cells or on ricin's enzymatic activity as assessed in vitro. Instead, all three compounds appear to function by blocking downstream stress-induced signaling pathways associated with the toxin-mediated apoptosis. PW66 virtually eliminated ricin-induced TNF-α secretion by J774A.1 macrophages and concomitantly blocked activation of the p38 MAPK and JNK signaling pathways. PW72 suppressed ricin-induced TNF-α secretion, but not p38 MAPK and JNK signaling. PW69 suppressed activity of the executioner caspases 3/7 in ricin toxin- and Shiga toxin 2-treated cells. While the actual molecular targets of the three compounds have yet to be identified, these data nevertheless underscore the potential of small molecules to down-regulate inflammatory signaling pathways associated with exposure to the RIP family of toxins.

## Introduction

Ricin, a heterodimeric glycoprotein found in the seeds of the castor bean plant (*Ricinus communis*), is an extraordinarily potent toxin. Ricin's enzymatic subunit (RTA) is an RNA N-glycosidase that irreversibly inactivates eukaryotic ribosomes through hydrolytic cleavage of a conserved adenosine residue within the sarcin-ricin loop (SRL) of 28S rRNA [1], [2]. Ricin's binding subunit (RTB) is a galactose- and N-acetylgalactosamine (Gal/GalNac)-specific lectin that mediates attachment, endocytosis, and trafficking of RTA from the plasma membrane to the endoplasmic reticulum (ER) [3]. Then through a process known as retro-translocation (or dislocation), RTA is threaded across the ER membrane and into the cytoplasm, [4], [5], [6], [7]. Once in the cytoplasm, RTA refolds into its enzymatically active conformation and initiates ribosome depurination at a rate estimated to exceed 1500/min [8].

As a direct consequence of rRNA depurination, RTA activates the so-called ribotoxic stress response (RSR) [9]. The RSR is associated with damage to 28S rRNA by a variety of toxic agents [10]. Through a mechanism that has yet to be fully elucidated, 28S rRNA damage stimulates cellular stress-activated protein kinases (SAPK), including p38 mitogen-activated protein kinase (p38 MAPK) and c-Jun N-terminal kinase (JNK) pathways. Activation of these and possibly other SAPKs by RTA leads to increased production of pro-inflammatory cytokines and apoptosis-mediated cell death [11], [12], [13]. The MAP3K, ZAK, has been identified as the being responsible for activating the p38 MAPK and JNK pathways in response to ricin [9], [11], [14].

Because ricin is a Category B biothreat agent, there is considerable interest in the identification of small molecules that block its cytotoxic effects [15]. In a recent report, we performed a cell-based, high-throughput screen (HTS) of >80,000 compounds from 17 commercially available chemical libraries [16]. In that initial screen, we identified a number of compounds that potentially interact with RTA's active site. In this study, we have screened an additional 118,000 compounds and have identified three new compounds that partially protect cells from the effects of ricin. Characterization of these compounds suggests they function not by interacting with ricin per se, but rather, by blocking stress-activated pathways associated with ricin-induced cell killing. This study is significant in that it contributes to an emerging body of evidence that suggests small molecule inhibitors of cell death and inflammation may have utility, alone or in combination with immunotherapeutics, as countermeasures against ricin and other related biothreat agents.

## Experimental Procedures

### Cell culture, reagents, and materials

Vero (CCL-81), J774A.1 (TIB-67), and THP-1 (TIB-202) cell lines were purchased from the American Type Culture Collection (ATCC; Manassas, VA). Vero and J774A.1 cells were routinely propagated in antibiotic-free Dulbecco's Modified Eagle Medium (DMEM) containing 10% fetal bovine serum (FBS) at 37°C in 95% air and 5% CO_2_, as described [16]. THP-1 cells were propagated in Roswell Park Memorial Institute (RPMI) medium with 10% FBS. Cell culture methods have been described previously [16].

Ricin (*Ricinus communis* agglutinin II), ricin-FITC, and RTA were obtained from Vector Laboratories (Burlingame, CA). Ricin was dialyzed against PBS to remove sodium azide prior to use. Shiga toxin 2 (Stx2) was a gift from Dr. Cheleste Thorpe (Tufts Medical Center, Boston MA). CellTiter-Glo™, control RNA (luciferase mRNA), and Bright-Glo™ Luciferase Assay System were purchased from Promega (Madison, WI). Luminescence was measured using an EnVision® (Perkin Elmer, Waltham, MA) or a SpectraMax® L Molecular Devices (Sunnyvale, CA) microplate luminometer. Goat anti-rabbit IgG conjugated to horseradish peroxidase (HRP) was purchased from Southern Biotech (Birmingham, AL). Nitrocellulose membranes were purchased from Biorad (Richmond, CA) while X-ray films were purchased from Krackeler Scientific Inc. (Albany, NY). Electrochemical luminescence (ECL) reagent was purchased from Pierce Scientific (Rockford, IL). Flow cytometry was performed using a FACSCalibur flow cytometer (BD Biosciences).

### HTS of small-molecule libraries

Primary screening of ∼118,700 pure compounds from the commercially available chemical libraries (Actimol TimTec, Bionet, ChemDiv, CEREP, Enamine, I.F. Lab, Maybridge, and Peakdale) was performed at the National Screening Laboratory for the Regional Centers of Excellence in Biodefense and Emerging Infectious Diseases (NSRB) at Harvard Medical School (Boston, MA) as previously described [16]. Briefly, Vero cells suspended in DMEM + 10% FBS (25 µl; ∼1.0×10^3^ cells) were seeded in 384-well opaque plates and incubated overnight at 37°C to allow the cells to adhere. Test compounds (100 nl; ∼30–90 µM final concentration) were then added to the assay wells. The cells were then incubated at 37°C for 1 h before the addition of 5 µl of ricin (∼0.08 nM final concentration). The cells were then incubated at 37°C for 48 h and cell viability was measured using CellTiter-Glo™. HTS data were analyzed essentially as described previously [16]. The Z-prime factor (Z′), a measure of robustness of an assay, for each test plate was determined as described [17]. Compounds were “cherry picked” if they met the following criteria: (i) were present on test plates with Z′ ≥0.5; (ii) gave a Z-score ≥2.0; and (iii) inhibited ricin-induced cytotoxity by ≥50%. Compounds that conferred 50–80% cell viability were considered moderate hits, whereas compounds that conferred ≥80% cell viability were classified as strong.

### Secondary and tertiary analysis of small molecule inhibitors

Secondary screens were performed as described for the primary screen but with one major modification: Cherry picked compounds (1.2 µl; 5 mg/ml in DMSO) from the primary screen were either diluted 10-fold in DMSO and then transferred (30–90 µM final concentration) to Vero cell assay plates using pin arrays, or transferred directly to Vero cells plates without dilution using PocketTips™. Tertiary analyses with graded concentrations of test compounds were performed essentially as described [16]. Briefly, Vero cells suspended in DMEM + 10% FBS (120 µl; ∼1.0×10^4^ cells) were seeded in a 96-well plate and incubated overnight at 37°C. 1 µl of 2-fold serially diluted (10 to 0.078 mM in DMSO) test compound was added in triplicate to the assay wells. An equal volume of DMSO was added in triplicate to the positive and negative control wells. The cells were incubated at 37°C for 30 min before 6.4 µl of ricin (∼0.2 nM final concentration) was added to the assay wells. Cells were then incubated at 37°C for 24 h before viability of the cells was measured. Purity of the test compounds (>90%) was confirmed by liquid chromatography mass spectrometry (LCMS) analysis and the final concentration of DMSO in each assay well was ∼0.8% v/v.

### Protein synthesis inhibition assay

Vero cells (5.0×10^5^ cells/ml), grown overnight in 24-well plates, were incubated with test compounds (25 µM) for 30 min before ricin (∼0.2 nM final concentration) treatment. Eight hours later, the growth medium was replaced with Met/Cys-free DMEM (Invitrogen, Carlsbad, CA) supplemented with 10 µCi/ml ^35^Met-^35^Cys (PerkinElmer, Boston, MA). Two hours later, the cells were washed with PBS and then treated with 5% of ice-cold trichloroacetic acid (TCA). Cell debris was scrapped from the plate and transferred to a scintillation vial with 5 ml of EcoScint (National Diagnostics, Atlanta, GA). Radioactivity was measured using Beckman LS 6500 Scintillation Counter (Ramsey, MN).

### In vitro translation (IVT) assay

The rabbit reticulocyte protein translation assays contained test compounds (3–94 µM), RTA (1.0 nM), *luc* mRNA (10 ng/ml), and DMSO (0.33% final) and were done as described previously [16], [18], [19], [20]. Retic Lysate IVT™ kit was purchased from Applied Biosystems/Ambion (Austin, TX).

### Inhibition of ricin binding to cell surfaces

FITC-labeled ricin (0.5 µg/ml; ∼8.0 nM) was incubated with varying concentrations (1 to 100 µM) of test compounds, DMSO (negative control) or galactose (30 mg/ml; ∼167 mM) for 30 min at 4°C before being applied to THP-1 cells (3×10^5^ ml). Following 30 min incubation at 4°C, the cells were washed to remove unbound ricin and then subjected to flow cytometry [21].

### Cytometric bead array (CBA)

The mouse inflammatory CBA kit (BD Biosciences, San Diego, CA) was used to measure Interleukin (IL)-6, IL-10, monocyte chemotactic protein-1 (MCP-1), interferon gamma (IFN-γ), tumor necrosis factor alpha (TNF-α), and IL-12p70 in cell supernatants [22].

### Activation of p38α MAPK and SAPK/JNK

J774A.1 cells were treated with test compounds (20 µM final concentration) or DMSO for 30 min before addition of ricin (0.2 nM final concentration). Cells were collected 6 h later for analysis of activated p38 MAPK using the nonradioactive kit (Cell Signaling Technology, Beverly, MA). For analysis of activated SAPK/JNK, cells were detached from the culture plates using a cell scraper and then collected by centrifugation. The resulting cell pellets were suspended in 1 vol of Laemmli sample buffer containing 5% β-mercaptoethanol, boiled, and subjected to SDS-PAGE and Western blotting with phospho-p38 MAPK and phospho-JNK-specific antibodies purchased from Cell Signaling Technology.

#### In vitro inhibition of p38-α MAPK activity

p38-α MAPK was immunoprecipitated from ricin-treated cell lysate (as described above) and then incubated for 5 min at RT with varying concentrations (5–40 µM) of the test compounds before the addition of ATP and ATF-2. The mixture was incubated for 30 min at 30°C, and then subjected to dot blot or Western blot analysis. ImageJ software was used for quantification of signal densities on exposed X-ray films.

#### Inhibition of caspases 3/7 activities

Vero cells (10,000 cells/well) were treated with test compounds (0.6–78.5 µM) for 30 min before treatment with ricin (0.2 nM) or Stx2 (1.5 nM) for 24 h or 48 h, respectively. Caspase 3/7 activities were measured using the ApoLive-Glo™ Multiplex Assay (Promega), according to the manufacturer's instruction.

## Results

### Identification of small-molecule inhibitors of ricin using a cell-based HTS

We used a Vero cell-based assay at the NSRB to screen more than 118,000 compounds in the Actimol TimTec, Bionet, ChemDiv, CEREP, Enamine, I. F. Lab, Maybridge, and Peakdale libraries for inhibitors of ricin toxin [16]. We identified approximately 400 that reduced ricin-induced cell death by ≥50% when assessed 48 h post-toxin treatment. In accordance with NSRB guidelines, we cherry-picked 356 compounds (0.3% of the total compounds screened) and retested each of them in a secondary screen at three different concentrations (33, 53, 83 µM) for the ability to reduce ricin cytotoxicity. Forty of the 356 compounds (“Group A”) demonstrated a dose-dependent inhibition of ricin cytotoxicity, while 70 compounds (“Group B”) demonstrated significant ricin inhibition at 33 µM, but much less so at higher concentrations, possibility because of limited solubility and/or cytotoxicity. The remaining cherry picks failed to inhibit ricin cytotoxicity and were therefore considered to be false positives.

### Identification of PW66, PW69 and PW72 from Group A compounds

**Figure 1:**
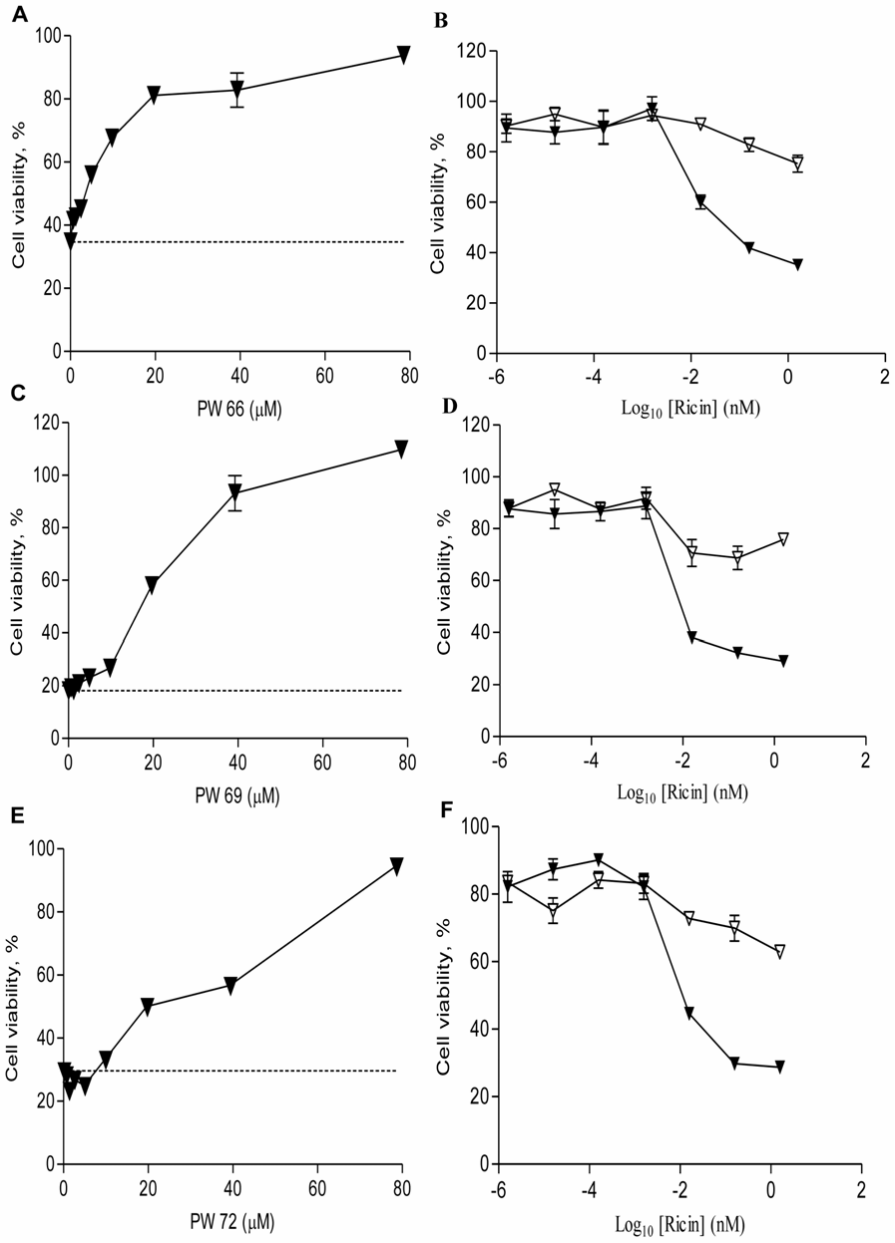
Compounds PW66, PW69 and PW72 inhibit ricin cytotoxicity. (Panels A, C, E) Vero cells were treated with ricin (0.2 nM; dashed lines) or pretreated with (A) PW66, (C) PW69, or (E) PW72 at the indicated concentrations for 30 min before ricin was added. Cell viability was measured at 24 h, as described in the Experimental Procedures. (Panels B, D, F) Vero cells were treated with indicated concentrations of ricin in the absence (solid symbols) or presence (open symbols) of (B) PW66, (D) PW69, and (F) PW72. Compounds were present at 25 µM. Each panel shows results of a representative experiment from three independent experiments that were done in triplicate and showed < 10% correlation of variation (% CV) for individual experiment.

**Figure 2:**
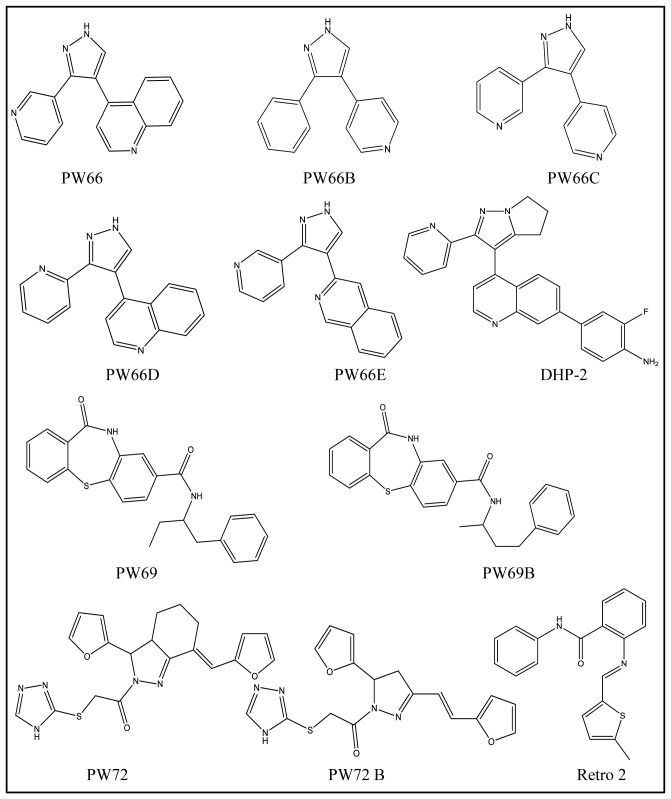
2D structure of compounds described in this work.

Thirty-two of the 40 Group A compounds were commercially available, and were therefore characterized in more detail. Upon retesting, three of the 32 compounds, designated PW66, PW69 and PW72, were of particular interest because of their effectiveness at inhibiting ricin cytotoxicity (EC_50_ values of 8, 23, 31 µM, respectively) in both Vero and J774A.1 (Table 1; Figures 1, 2; Fig. S1). In fact, PW66, PW69 and PW72 were each more effective than Retro-2 at reducing toxin-induced death when assessed at 24 h (Fig. S2). Retro-2 is a recently identified small molecule that blocks retrograde transport of ricin (and Shiga toxin) between early endosomes and the TGN [23], [24]. Compounds PW66, PW69 and PW72 were themselves not toxic to Vero cells, even at concentrations ≥100 µM (Table S1). For these reasons, we chose to investigate them in greater detail. The remaining 29 commercially available compounds in Group A are being pursued in a separate study (P. Wahome and N. Mantis, manuscript in preparation).

**Table 1:** Compounds described in this study.

Compound	Systematic Name	ID	Vendor^a^	EC_50_ (µM)
PW66	4-(5-pyridin-3-yl-1H-pyrazol-4-yl) quinoline	4056795*^b^*	Peakdale	8
PW66B	4-(5-Phenyl-1H-pyrazol-4-yl)pyridine	4378208*^c^*	Peakdale	10
PW66C	3-(5-Phenyl-1H-pyrazol-4-yl)pyridine	2568949*^c^*	Peakdale	36
PW66D	4-(3-Pyridin-2-yl)-1H-pyrazol-4-ylquinoline	3000655*^d^*	Peakdale	9
PW66E	2-[5-(3-Pyridinyl)-1H-pyrazol-4-yl]quinoline	4285370*^c^*	Peakdale	9
PW69	6-oxo-N-(1-phenylbutan-2-yl)-5H-benzo[b][1], [4] benzothiazepine-3-carboxamide	6623219*^b^*	ChemDiv	23
PW69B	6-oxo-N-(4-phenylbutan-2-yl)-5H-benzo[b][1], [4] benzothiazepine-3-carboxamide	20885127*^b^*	ChemDiv	22
PW72	1-[3-(furan-2-yl)-7-(furan-2-ylmethylidene)-3a,4, 5,6-tetrahydro-3H-indazol-2-yl]-2-(1H-1,2,4-triazol-5-ylsulfanyl)ethanone	4342949*^b^*	Enamine	31
PW72B	1-[3-(furan-2-yl)-5-[(E)-2-(furan-2-yl)ethenyl]-3, 4-dihydropyrazol-2-yl]-2-(1H-1,2,4-triazol-5-yl sulfanyl) ethanone	16358890*^b^*	Enamine	26

a Source of the compound; *^b^*PubChem ID; *^c^*Chemspider ID; *^d^*Vendor ID.

We next examined the inhibitory activity of select analogs of compounds PW66, PW69 and PW72 (Table 1; Figure 2). Three analogs of PW66 (B, D and E) had EC_50_s similar to PW66. Analogue C, on the other hand, was 4-fold less effective than PW66, possibly due to the proximity of two nitrogen atoms in the pendant groups (pyridines) of compound. Compounds PW69B and PW72B had ricin inhibitory activities similar to PW69 and PW72, respectively (Table 1).

Finally, using the Vero cell cytoxicity assay, we tested PW66, PW69 and PW72 in various combinations and concentrations in order to determine whether a mixture of the compounds would be more effective than individual compounds at reducing ricin-induced cell death. Surprisingly, preliminary checkerboard analysis did not reveal any evidence of synergy or even additivity between the compounds (data not shown). While our initial supposition based on these studies is that the compounds are not more effective when combined, a further more detailed analysis using models such as Bliss independence or Loewe additivity is required before such a conclusion can be fully substantiated.

### PW66, PW69, and PW72 act downstream of ricin-induced protein synthesis inhibition

To begin to define the mechanism by which PW66, PW69 and PW72 reduce ricin-induced cytotoxicity, we first examined each of the compounds for the ability to block RTA-mediated protein synthesis inhibition. In a Vero cell assay, ricin treatment reduced cellular protein synthesis levels by ≥60% at 8 h. The addition of 25 µM PW66, PW69 or PW72 before and during toxin treatment did not affect ricin's capacity to inhibit protein synthesis in Vero cells (Fig. S3), indicating that the three compounds do not interfere with ricin entry, intracellular trafficking, retrotranslocation, or ribosome inactivation. This is in contrast to Retro-2, which we confirmed is partially effective at blocking ricin's capacity to arrest protein synthesis (data not shown). We did note, however, that PW66, PW69 or PW72 each had an impact on baseline protein synthesis in this assay even though the compounds themselves were not cytotoxic to Vero cells (Table S1; Fig. S3).

To determine whether PW66, PW69 or PW72 have any direct effect on RTA or the host translational machinery, each of the compounds were tested in an IVT assay [16], [18], [19], [20]. We found that none of the compounds (3–94 µM) had any demonstrable impact on RTA's ability to arrest protein synthesis or on translation itself (Fig. S4; data not shown). From these studies we conclude that PW66, PW69 or PW72 must interfere with ricin's cytotoxic effects at step(s) downstream of ribosome arrest.

### PW66 and PW72 (but not PW69) reduce ricin-induced TNF-α secretion by macrophages

**Figure 3:**
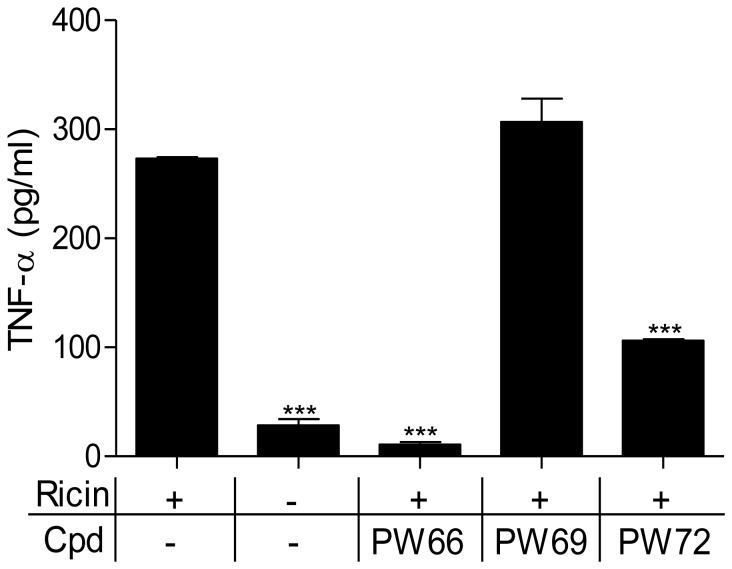
Compounds PW66 and PW72 inhibit TNF-α secretion by ricin-treated murine macrophages. J774A.1 cells were treated with ricin (0.2 nM) and PW66, PW69 or PW72 for 24 h. Soluble TNF-α levels were quantified in the growth medium as described in the Experimental Procedures. Shown are results of a representative experiment that was done in duplicate and showed <10% variation. One-way ANOVA with Dunnett's multiple comparison test was performed using GraphPad Prism version 5.00 for Windows (GraphPad software, San Diego, CA) USA. *p* values less than 0.05 were considered statistically significant (***).

The fact that PW66, PW69 and/or PW72 did not interfere with protein synthesis inhibition per se, led us to hypothesize that they suppress toxin-induced SAPK activation and/or apoptosis. Induction of the RSR by RTA, for example, triggers the p38 MAPK and JNK pathways, resulting in the secretion of the pro-inflammatory cytokine TNF-α [10], [12], [25], [26]. To examine the influence of each of the compounds on TNF-α production, J774A.1 cells were treated with the PW66, PW69 or PW72 for 30 min prior to ricin treatment. TNF-α levels were then measured in culture supernatants 24 h later. We found that ricin treatment alone resulted in ∼10-fold increase in TNF-α in cell supernatants (Fig. 3). PW66 completely blocked ricin-induced TNF-α production, whereas PW72 reduced TNF-α levels by >50%. PW69, in contrast, did not impact ricin-induced TNF-α production. These data suggest that PW66 and PW72 likely block activation of the p38 MAPK and/or JNK signaling pathways in response to ricin treatment.

### PW66 inhibits activation of the p38 MAPK and JNK pathways

**Figure 4:**
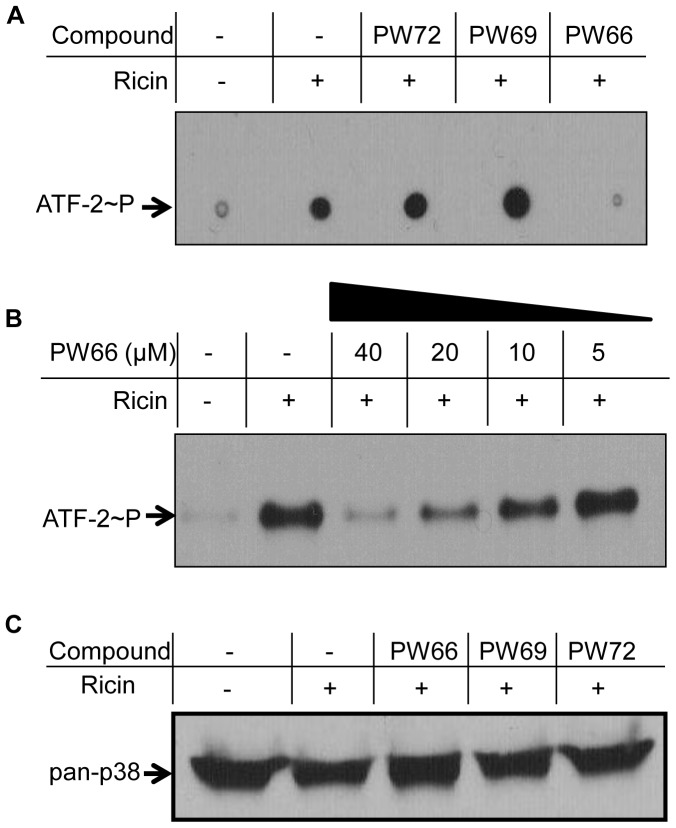
Compound PW66 interferes with activation of p38 MAPK in ricin-treated cells. (A) J774A.1 cells were treated with ricin (0.2 nM) with or without indicated compounds for 6 h before cells were lysed. Phospho-p38 MAPK was immunoprecipitated from cell lysates and used in an *in vitro* ATF-2 phosphorylation assay, as described in the Experimental Procedures. Shown are the results of a dot-blot analysis. (B) J774A.1 cells were treated with ricin (0.2 nM) with or without PW66 at indicated concentrations as shown in Panel A, except that ATF-2 was subjected to SDS-PAGE and Western blotting. (C) Western blot analysis of total p38 MAPK from ricin- or ricin + compound-treated cells, as indicated. Shown are results of representative experiments from three or more independent experiments.

We used a coupled immunoprecipitation/ATF-2-phosphorylation assay to determine whether PW66 and/or PW72 interfere with ricin-induced activation of p38 MAPK. As expected, treatment of J774A.1 cells with ricin alone resulted in a significant increase in the endogenous levels of phospho-p38 MAPK, as evidenced by the high signal intensity of phosphorylated ATF-2 (Fig. 4A). No such activation of ATF-2 was evident when cells were treated with PW66. Indeed, PW66 demonstrated a dose-dependent capacity to inhibit ricin-induced phospho-p38 MAPK (Fig. 4B). In contrast, PW69 and PW72 did not reduce (but, in fact, marginally enhanced) activation of p38 MAPK (Fig. 4A). None of the compounds had any effect on the endogenous levels of unphosphorylated p38 MAPK in Vero cells (Fig. 4C).

To examine whether PW66 influences p38 MAPK phosphotransferase activity, we immunoprecipitated p38 MAPK from ricin-treated cells and then performed an *in vitro* ATF-2 phosphorylation reaction in the presence or absence of PW66. The addition of PW66 did not alter p38 MAPK phosphotransferase activity *in vitro* (data not shown), indicating that PW66 likely prevents p38 MAPK phosphorylation by an upstream kinase, and not by interfering with p38 MAPK's activity per se.

**Figure 5:**
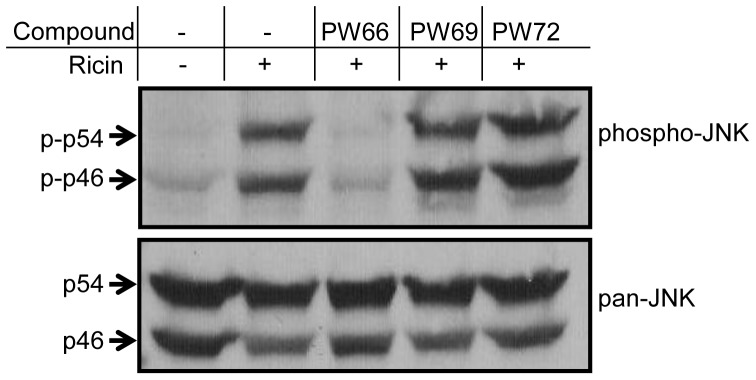
Compound PW66 interferes with activation of SAPK/JNK in ricin-treated cells. J774A.1 cells were treated with ricin (0.2) in the presence or absence of indicated compounds for 6 h before cells were lysed and subjected to SDS-PAGE and Western blot analysis with (A) phospho-JNK or (B) JNK specific antibodies. Shown are results of representative experiments from three or more independent experiments.

To test whether PW66 had a similar inhibitory effect on activation of the JNK pathway, total cell lysates were probed by Western blot with antibodies specific for phospho-JNK. As expected, ricin alone caused an increase in phospho-JNK levels (Fig. 5). Treatment of cells with compound PW66 completely blocked ricin-induced activation of JNK, whereas neither PW69 nor PW72 had any effect on phospho-JNKs levels (Fig. 5, upper panel). None of the compounds had notable effect on the endogenous levels of unphosphorylated JNK in Vero cells (Fig. 5, lower panel).

### Compounds PW69 inhibit caspases 3/7 activities

**Figure 6:**
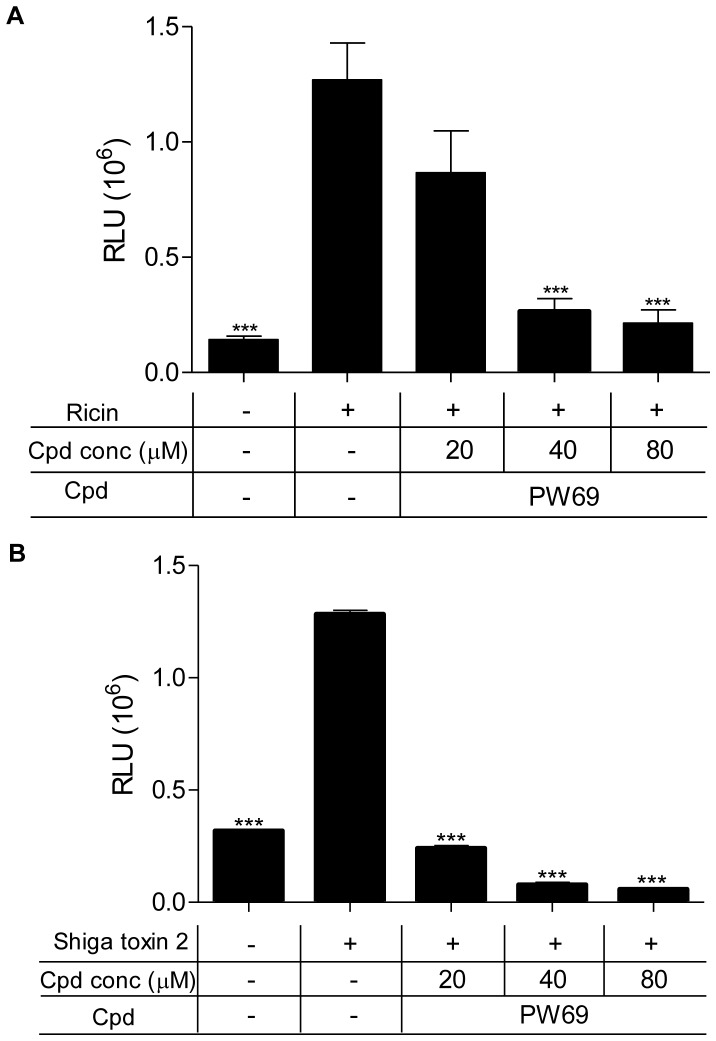
Compound PW69 interferes with the activity of caspases 3/7. Vero cells were treated with (A) ricin (0.2 nM) or (B) Stx2 (1.5 nM) in the absence or presence of indicated concentrations of PW69. Cells were then lysed and incubated with caspases 3/7 substrates, as described in the Experimental Procedures. The results are from a representative experiment that was performed in triplicate and showed <10% variation. One-way ANOVA with Dunnett's multiple comparison test was performed and *p* values less than 0.05 were considered statistically significant (***).

Since neither PW69 nor PW72 blocked ricin-induced SAPK pathways, we postulated that they might act on another aspect of toxin-mediated cell death, such as activation of executioner caspases 3 and 7 [27]. To this end, Vero cells were pretreated with PW69, PW72 or PW66 for 30 min prior to ricin exposure. Activities of executioner caspases 3 and 7 were determined using a luciferase-based substrate. We found that ricin treatment alone resulted in a ∼10 fold increase in caspase 3/7 activities, as compared to mock-treated cells (Fig. 6A). Neither PW66 nor PW72 significantly influenced caspase activation. PW69, on the other hand, demonstrated a dose-dependent reduction in caspase 3/7 activities (Fig. 6A; data not shown). Moreover, PW69 interfered with toxin-induced DNA fragmentation in J774A.1 cells, while PW66 and PW72 did not (data not shown). To examine whether PW69 could also interfere with the activity of caspases 3 and 7 in Vero cells treated with a different inducer of apoptosis, cells were treated with Stx2. PW69 exhibited a dose-dependent inhibition of Stx2-induced caspase (Figure 6B). These data suggest that PW69 interferes with ricin- and Stx2-induced apoptosis.

## Discussion

Two very different experimental screening strategies have been employed over the past two decades in an effort to identify small molecule inhibitors of ricin (and Shiga toxins), with very different outcomes. On the one hand, virtual library screening has led to the identification of three broad classes of active site (or near active site) inhibitors [15]. For example, virtual screening identified pteroic acid (PTA), a small molecule that was subsequently shown by X-ray crystallography to bind RTA's active site with nearly perfect complimentarity [28]. Subsequent rational design and medicinal chemistry strategies have been employed to develop a diversified collection of pterin-based compounds, like 7-carboxy pterin (7CP), that make additional contacts with RTA and that improve relative IC_50_s [29].

Cell-based screening strategies, on the other hand, have led to the identification of small molecules that (partially) protect cells from ricin-induced death. The compounds identified to date by this strategy do not act on the toxin per se, but rather interfere with cellular processes required for toxin intracellular transport or trafficking [15]. Using a cell-based HTS, Haslam and colleagues identified two compounds, 75 and 134, from the known bioactive and ChemDiv3 chemical libraries [30]. Compound 75 inhibited intracellular transport of Shiga toxin 1 (Stx1) to perinuclear recycling endosomes, while compound 134 inhibited transport of Stx1 at a post-recycling endosome stage. Stechmann and colleagues identified Retro 2 from a screen of ∼16,500 compounds in the ChemBridge library [24].

We have now completed a high-throughput, cell-based screen of more than 118,000 compounds from diverse chemical libraries and identified three compounds, PW66, PW69 and PW72 with varying capacities to reduce ricin-induced cell death. Like the other cell-based screens, none of the compounds we identified appear to act on ricin directly. Rather, PW66, PW69 and PW72 apparently act by blocking toxin-induced activation of one or more SAPK or pro-apoptotic pathways. Collectively, PW66, PW69 and PW72 constitute a class of “downstream” ricin inhibitors, in contrast to Retro 2 and Compounds 75/134, which interfere with trafficking of ricin from the plasma membrane to the ER.

Our data are consistent with PW66 functioning as an inhibitor of ZAK (also known as MRK and MLKT-α), the upstream MAP3K responsible for activating the p38 MAPK and JNK pathways in response to ricin and other ribotoxic stressors [9], [11], [14]. Jandhyala and colleagues demonstrated that treatment of HCT-8 or Vero cells with the ZAK inhibitor DHP-2 (200 nM) blocked ricin-induced IL-8 production and suppressed activation of both p38 MAPK and JNK pathways [11]. In this study we demonstrated that PW66 is similar to DHP-2 in that it suppressed ricin-induced TNF-α in J774 cells and p38 MAPK and JNK pathways in Vero cells. Although we have not demonstrated that PW66's target is in actually ZAK, it is interesting to note that compound PW66 is structurally related to DHP-2, an aryl-substituted dihydro-pyrrolopyrazole quinoline [31]. The fact that PW66 was identified from among the more than 118,000 compounds screened in this study attests a possible central role of ZAK (or a ZAK-like MAP3K) in orchestrating ricin-induced stress-activated signaling pathways.

It is not immediately obvious how ZAK inhibitors like DHP-2 (and possibly PW66) interfere with ricin-induced cell death. On the one hand, there is considerable evidence for “cross talk” between pathways involved in inflammation and apoptosis, particularly via the p38 MAPK pathway [12], [26], [32], [33], [34]. Higuchi et al, for example, reported a decrease in ricin-induced apoptosis of murine macrophage (e.g., RAW 264.7) when the cells were treated with a specific p38 MAPK inhibitor [12]. Magun and colleagues, on the other hand, recently reported that ricin-mediated release of the pro-inflammatory cytokine IL-1β via the NALP3 inflammasome in bone marrow-derived macrophages is enhanced, rather than suppressed, by inhibition of SAPK phosphorylation [35]. Because ricin triggers the SAPKs, as well as other proinflammatory pathways, sorting out the relevance of specific inhibitors in dampening ricin's pathophysiology at the cellular and tissue levels awaits comprehensive animal studies.

The mechanism(s) by which PW69 and PW72 limit ricin-induced cell killing are yet to be determined. To our knowledge, neither compound is structurally related to any previously described inhibitors of ricin or apoptosis [15], [16], [20], [24], [30], [36]. PW72 was effective at blocking ricin-induced TNF-α release by J774 cells, but did not suppress signaling via p38 MAPK or JNK. Based on this result, we hypothesize that PW72 must interfere with TNF-α synthesis, intracellular trafficking, and/or proteolytic release from the cell surface [37]. PW69, on the other hand, blocked ricin-induced up-regulation of executioner caspases 3 and 7, strongly suggesting that this compound works by interfering with cellular progression to apoptosis. The fact that PW66, PW69 and PW72 all appear to partially protect cells from ricin-induced killing by targeting host proteins or host pathways, provides further support to the idea that inhibitors of toxin-induced SAPK pathways could be utilized, alone or in conjunction with immunotherapies, to mitigate inflammatory responses initiated by ricin or related RIPs in systemic and mucosal compartments [9].

## Supporting Information

Figure S1 **Compounds PW66, PW69 and PW72 inhibit ricin cytotoxicity.** Vero cells were treated with ricin (0.2 nM; dashed lines) or pretreated with PW66 (open circles), PW69 (open squares), or PW72 (open triangles) at the indicated concentrations for 30 min before ricin was added. Cell viability was measured at (A) 48 hr or (B) 72 hr as described in the Experimental Procedures. Each panel shows results of a representative experiment from three independent experiments that were done in triplicate and showed <10% correlation of variation (% CV) for individual experiment.
(TIF)

Figure S2 **Inhibition of ricin cytotoxicity by Retro 2.** Vero cells were treated with ricin (0.2 nM; dashed lines) or pretreated with Retro 2 (filled triangles) at the indicated concentrations for 30 min before ricin was added. Cell viability was measured at 24 hr, as described in the Experimental Procedures. Shown are results of a representative experiment from three independent experiments that were done in triplicate and showed <10% correlation of variation (% CV) for individual experiment.
(TIF)

Figure S3 **Compounds PW66, PW69, and PW72 do not inhibit the effect of ricin on protein biosynthesis.** Vero cells were treated with ricin (0.2 nM) or pretreated with 25 µM of PW66, PW72, PW69 or Retro 2 for 30 min before an aliquot of the growth medium (DMEM + 10% FBS) with or without ricin was added. The cells were incubated for 8 hr at 37°C, pulsed with10 µCi/ml ^35^Met-^35^Cys for 2 hr, washed, treated with 5% TCA, and the activity of incorporated radioisotopes was measured as described in the Experimental Procedures. Shown are results of a representative experiment that was done in quadruplicate and showed <10% correlation of variation (% CV) for individual experiment.
(TIF)

Figure S4 **Compounds PW66, PW69, and PW72 do not inhibit the enzymatic activity of RTA or significantly impact protein synthesis** ***in vitro*** **.** Individual test compound (94 µM) or DMSO (carrier solvent for compounds) was mixed with RTA (1.6 nM) or PBS (pH 7.4) and then added to an *in vitro* translation reaction in which luciferase mRNA was present as template. Translation of the luciferase mRNA was determined by addition of Bright-Glo™ substrate and measurement of light emission with a luminometer, as described in the Experimental Procedures. Shown are results of a representative experiment that was done in duplicate and showed <10% correlation of variation (%CV) for individual experiment.
(TIF)

Table S1 **Viability of Vero cells treated with varying concentrations of PW66, PW69, PW72 or Retro 2.**
(DOC)
