# Supplementary figures and images for: Identification of Small Molecules That Suppress Ricin-Induced Stress-Activated Signaling Pathways
Source: PLoS One. 2012 Nov 1;7(11):e49075. doi: 10.1371/journal.pone.0049075 (PMC3486792; doi:10.1371/journal.pone.0049075)

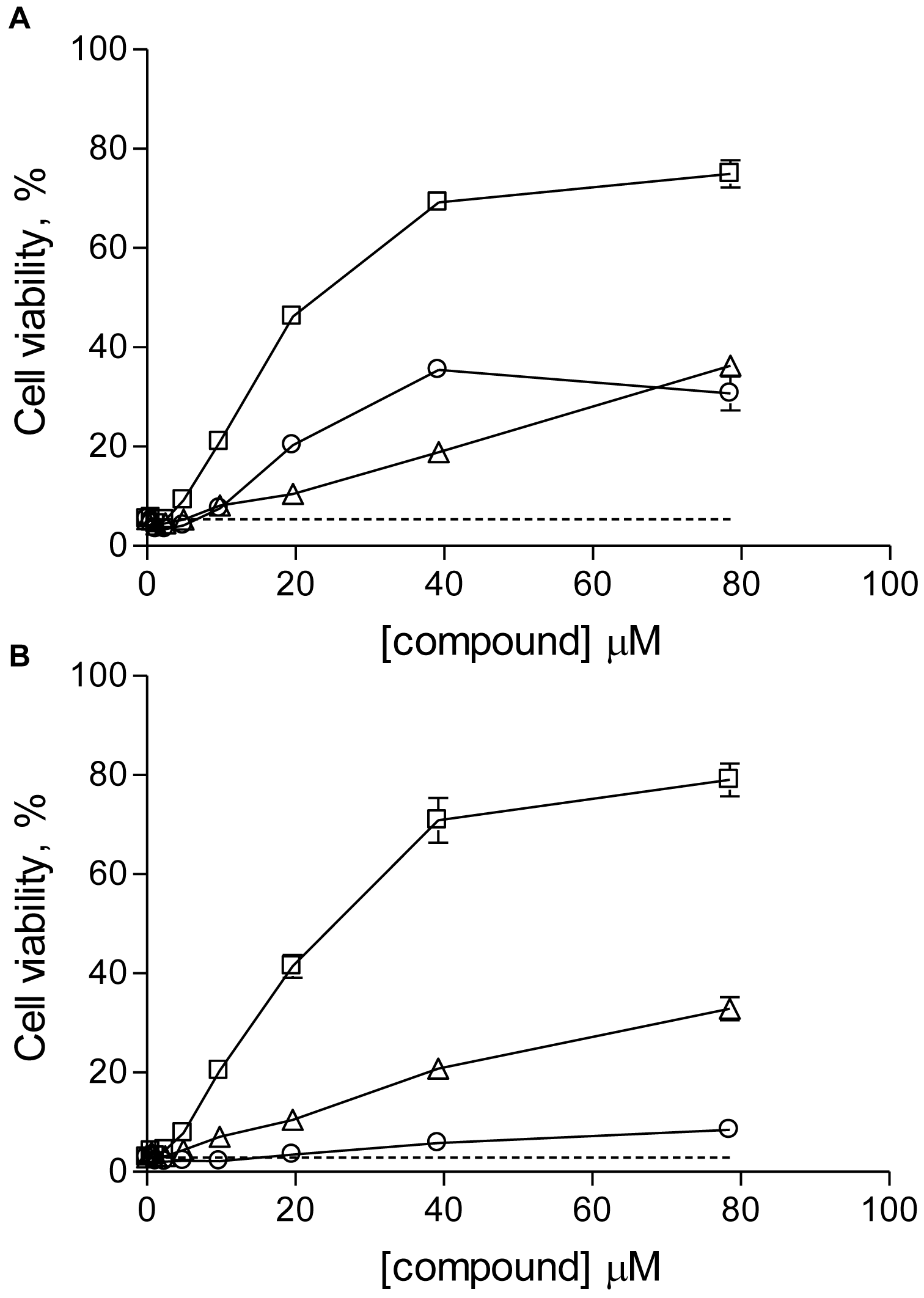

Supplement: Figure S1 — Compounds PW66, PW69 and PW72 inhibit ricin cytotoxicity. Vero cells were treated with ricin (0.2 nM; dashed lines) or pretreated with PW66 (open circles), PW69 (open squares), or PW72 (open triangles) at the indicated concentrations for 30 min before ricin was added. Cell viability was measured at (A) 48 hr or (B) 72 hr as described in the Experimental Procedures. Each panel shows results of a representative experiment from three independent experiments that were done in triplicate and showed <10% correlation of variation (% CV) for individual experiment. (TIF) [file pone.0049075.s001.tif]

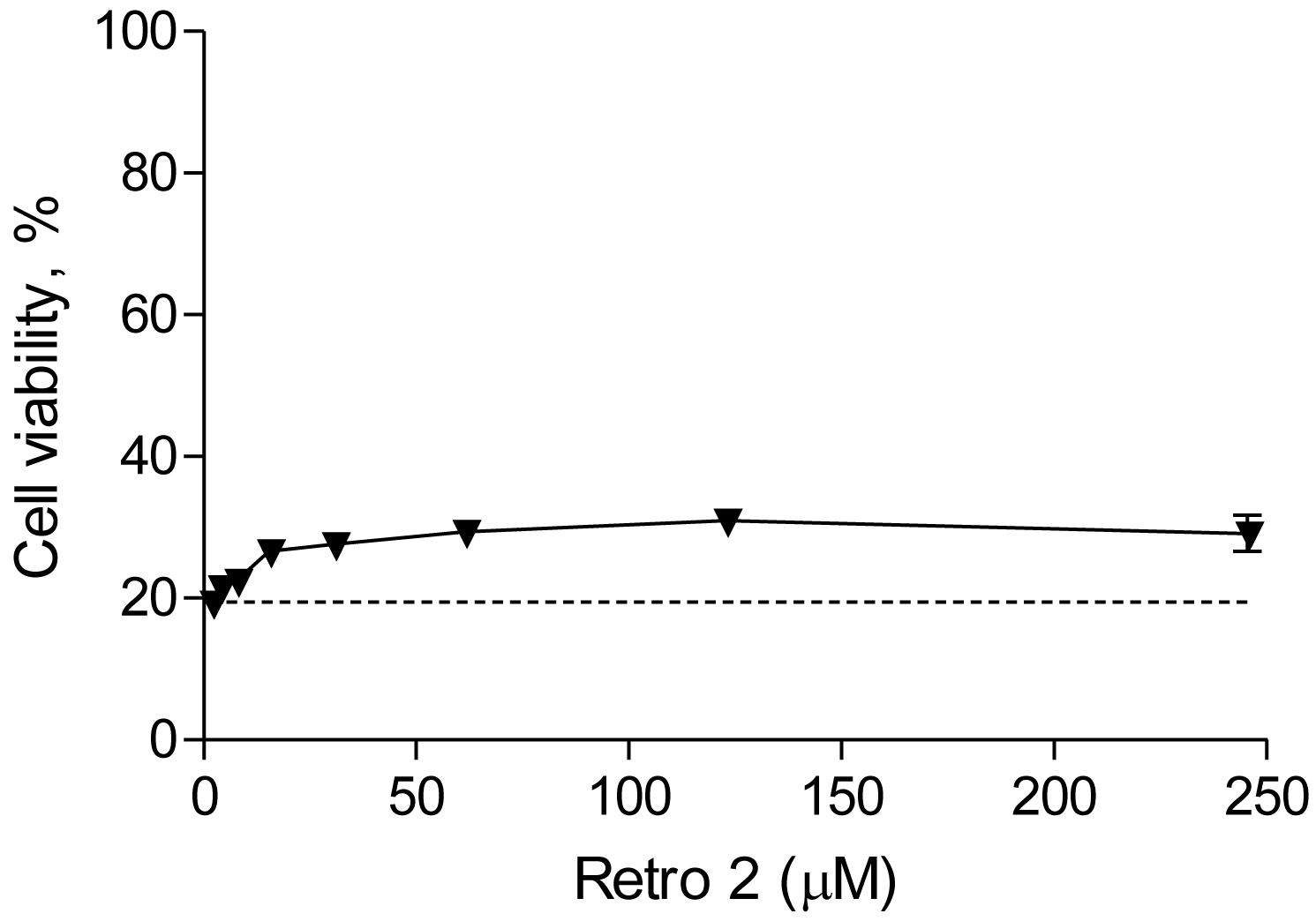

Supplement: Figure S2 — Inhibition of ricin cytotoxicity by Retro 2. Vero cells were treated with ricin (0.2 nM; dashed lines) or pretreated with Retro 2 (filled triangles) at the indicated concentrations for 30 min before ricin was added. Cell viability was measured at 24 hr, as described in the Experimental Procedures. Shown are results of a representative experiment from three independent experiments that were done in triplicate and showed <10% correlation of variation (% CV) for individual experiment. (TIF) [file pone.0049075.s002.tif]

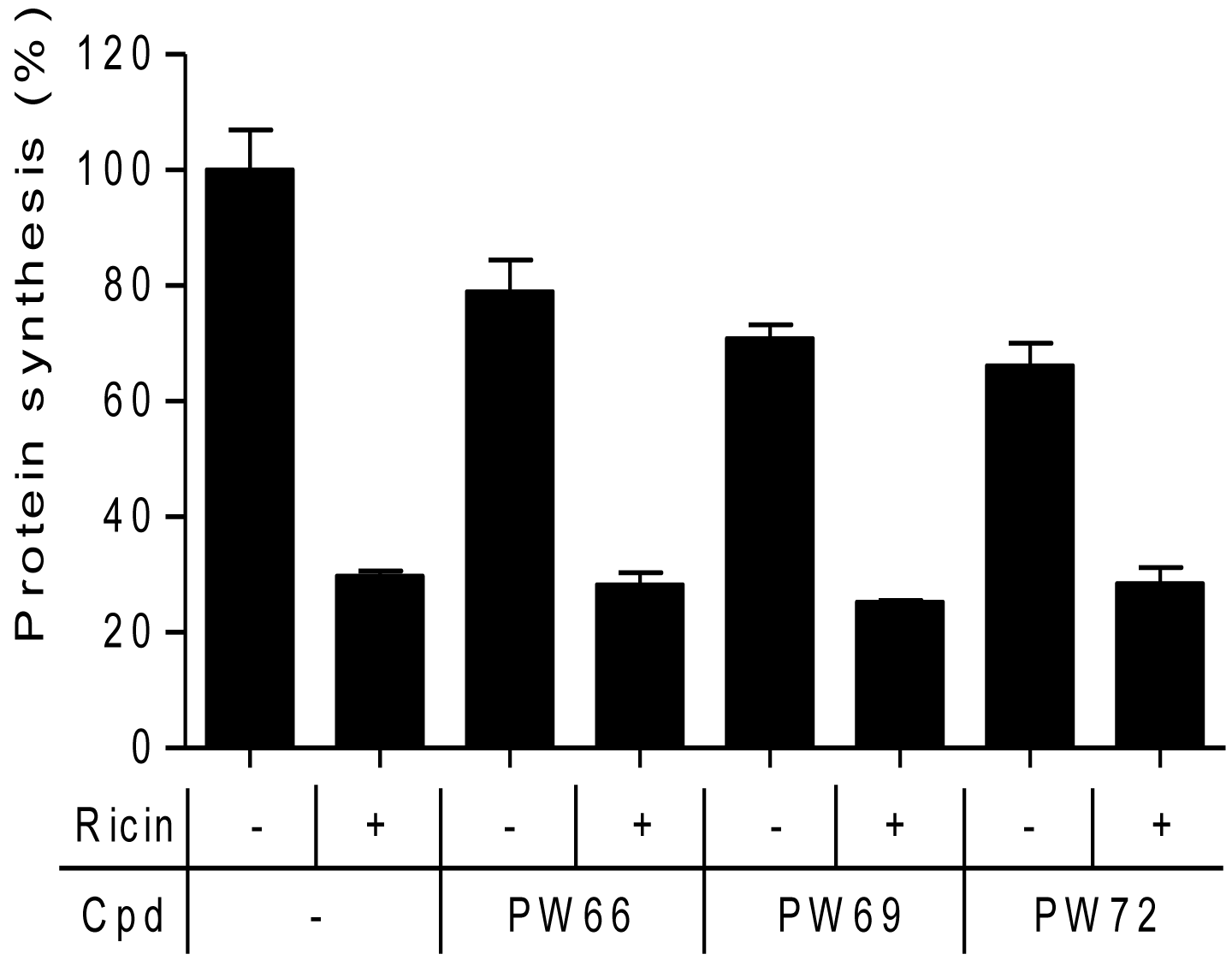

Supplement: Figure S3 — Compounds PW66, PW69, and PW72 do not inhibit the effect of ricin on protein biosynthesis. Vero cells were treated with ricin (0.2 nM) or pretreated with 25 µM of PW66, PW72, PW69 or Retro 2 for 30 min before an aliquot of the growth medium (DMEM + 10% FBS) with or without ricin was added. The cells were incubated for 8 hr at 37°C, pulsed with10 µCi/ml 35Met-35Cys for 2 hr, washed, treated with 5% TCA, and the activity of incorporated radioisotopes was measured as described in the Experimental Procedures. Shown are results of a representative experiment that was done in quadruplicate and showed <10% correlation of variation (% CV) for individual experiment. (TIF) [file pone.0049075.s003.tif]

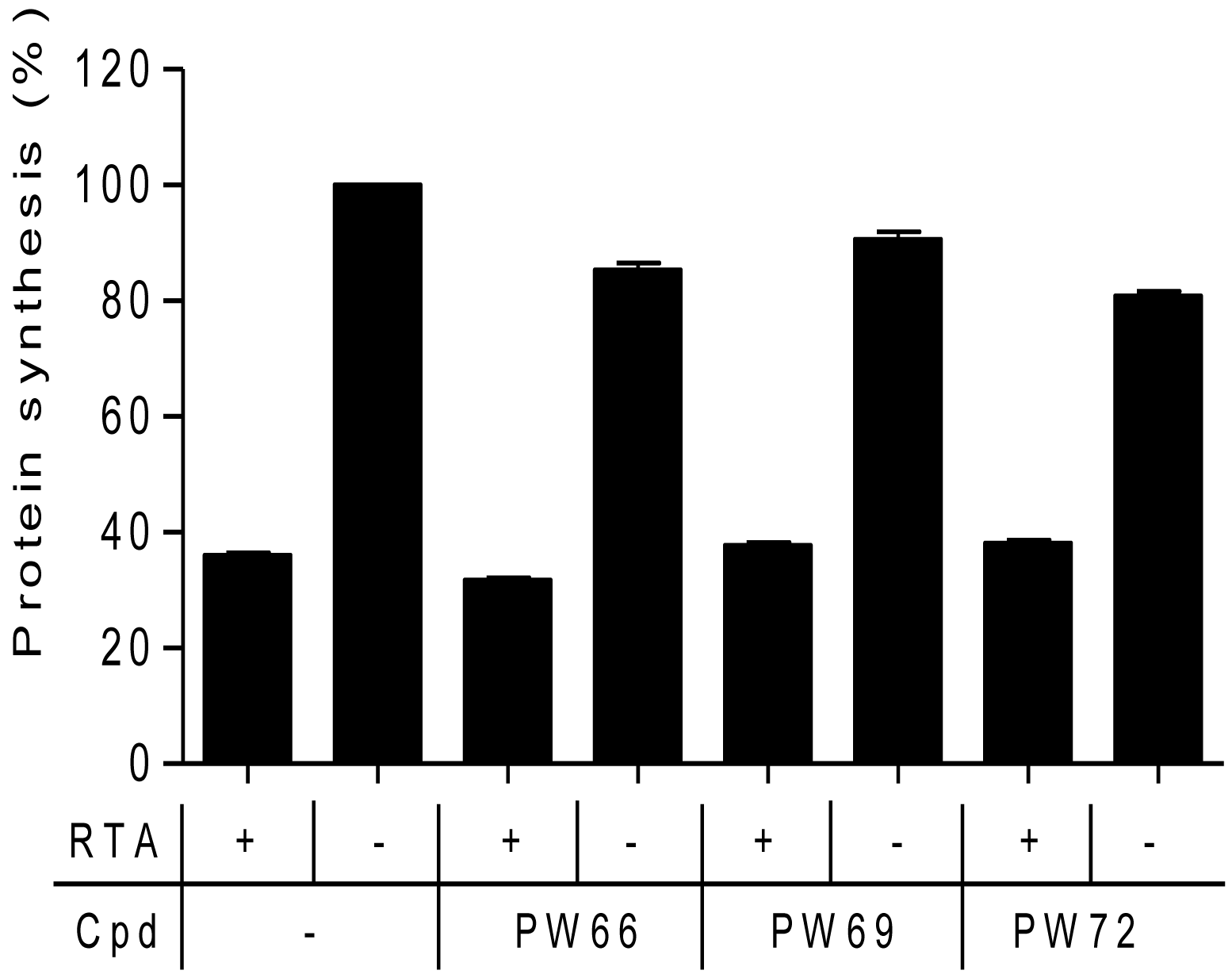

Supplement: Figure S4 — Compounds PW66, PW69, and PW72 do not inhibit the enzymatic activity of RTA or significantly impact protein synthesis in vitro . Individual test compound (94 µM) or DMSO (carrier solvent for compounds) was mixed with RTA (1.6 nM) or PBS (pH 7.4) and then added to an in vitro translation reaction in which luciferase mRNA was present as template. Translation of the luciferase mRNA was determined by addition of Bright-Glo™ substrate and measurement of light emission with a luminometer, as described in the Experimental Procedures. Shown are results of a representative experiment that was done in duplicate and showed <10% correlation of variation (%CV) for individual experiment. (TIF) [file pone.0049075.s004.tif]
