# Supplementary material for: Identification of Small Molecules That Suppress Ricin-Induced Stress-Activated Signaling Pathways
Source: PLoS One. 2012 Nov 1;7(11):e49075. doi: 10.1371/journal.pone.0049075 (PMC3486792; doi:10.1371/journal.pone.0049075)
Supplement: Table S1 — Viability of Vero cells treated with varying concentrations of PW66, PW69, PW72 or Retro 2. (DOC) [file pone.0049075.s005.doc]

| **Table S1.** **Viability of Vero cells treated with varying concentrations of PW66, PW69, PW72 or Retro 2.** | | | | |
| --- | --- | --- | --- | --- |
| **Compound** | **Concentration (μM)** | | | |
|  | **10** | **20** | **40** | **80** |
| PW66 | 99 (3.9)*^a^* | 91 (2.98) | 88 (3.23) | 85 (4.54) |
| PW69 | 84 (3.2) | 87 (7.0) | 94 (1.5) | 91 (6.4) |
| PW72 | 80 (6.6) | 80 (7.8) | 70 (11.3) | 78 (0.00) |
| Retro 2 | 103 (13.1) | 100 (0.5) | 79 (3.5) | 82 (10.2) |

*^a^,*

Shown in parenthesis is the correlation of variation (CV) value for a representative experiment done in triplicate.
